# A new MR-SAD algorithm for the automatic building of protein models from low-resolution X-ray data and a poor starting model

**DOI:** 10.1107/S2052252517017961

**Published:** 2018-01-25

**Authors:** Pavol Skubák, Demet Araç, Matthew W. Bowler, Ana R. Correia, Andre Hoelz, Sine Larsen, Gordon A. Leonard, Andrew A. McCarthy, Sean McSweeney, Christoph Mueller-Dieckmann, Harm Otten, Gabriel Salzman, Navraj S. Pannu

**Affiliations:** a Department of Biophysical Structural Chemistry, Leiden University, Einsteinweg 55, 2333 CC Leiden, The Netherlands; b Department of Biochemistry and Molecular Biology, The University of Chicago, Chicago, IL 60637, USA; c European Molecular Biology Laboratory, Grenoble Outstation, 71 Avenue des Martyrs, 38000 Grenoble, France; d Division of Chemistry and Chemical Engineering, California Institute of Technology, 1200 East California Boulevard, Pasadena, CA 91125, USA; e Department of Chemistry, University of Copenhagen, Universitetsparken 5, DK-2100 Copenhagen, Denmark; f European Synchrotron Radiation Facility, 71 Avenue des Martyrs, CS 40220, 38043 Grenoble, France; g Department of Photon Sciences, Brookhaven National Laboratory, Upton, NY 11973-5000, USA

**Keywords:** low resolution, X-ray crystallography, single-wavelength anomalous diffraction, multivariate statistics, model bias, structure determination, membrane proteins, refinement, multi-protein complexes

## Abstract

Determining macromolecular structures from X-ray data with resolution worse than 3 Å remains a challenge. Even if a related starting model is available, its incompleteness or its bias together with a low observation-to-parameter ratio can render the process unsuccessful or very time-consuming. Yet, many biologically important macromolecules, especially large macromolecular assemblies, membrane proteins and receptors, tend to provide crystals that diffract to low resolution. A new algorithm to tackle this problem is presented that uses a multivariate function to simultaneously exploit information from both an initial partial model and low-resolution single-wavelength anomalous diffraction data. The new approach has been used for six challenging structure determinations, including the crystal structures of membrane proteins and macromolecular complexes that have evaded experts using other methods, and large structures from a 3.0 Å resolution F_1_-ATPase data set and a 4.5 Å resolution SecYEG–SecA complex data set. All of the models were automatically built by the method to *R*_free_ values of between 28.9 and 39.9% and were free from the initial model bias.

## Abstract 2

A new algorithm automatically determines the structures of large macromolecules of unknown fold from low-resolution single-wavelength anomalous X-ray data and a partial model that failed with other methods.

## 1. Introduction   

Hardware and software advances have contributed greatly to the deposition of over 100 000 crystal structures in the Protein Data Bank (PDB; Berman *et al.*, 2003 ▸). Yet, despite the rapid growth in the number of macromolecular crystal structures determined, as of September 2017 over 93% of PDB depositions relate to diffraction data collected to a resolution better than 3.0 Å, while over 98% of depositions relate to diffraction data collected to resolutions better than 3.5 Å. Valuable information can be obtained from low-resolution structures (Schröder *et al.*, 2010 ▸). For example, biologically important macromolecules, including large macromolecular assemblies, membrane proteins and receptors, tend to result in crystals that diffract relatively poorly. Unfortunately, solving crystal structures from low-resolution data is difficult and time-consuming and can fail. While the extensive number of entries in the PDB means that a starting model for such structures can often be obtained by molecular replacement (MR), a poor observation-to-parameter ratio and potential model bias from the MR solution can complicate subsequent model building and refinement. Observation-to-parameter ratios can be improved by combining incomplete MR model information with information from anomalously scattering sulfur, phosphate, halogen or metal atoms or selenomethionine residues engineered into the protein to solve the structure, a technique referred to as MR-SAD (see, for example, Baker *et al.*, 1995 ▸; Schuermann & Tanner, 2003 ▸; Panjikar *et al.*, 2009 ▸). However, a poor starting MR model, low-resolution data, a weak anomalous signal, radiation damage or crystal anomalies such as translational noncrystallographic symmetry can often combine to prevent structure solution.

Previously, we have shown that a SAD ‘combined’ protocol (Skubák & Pannu, 2013 ▸) can substantially improve the success rate and quality of models built from an experimental SAD map. By definition, an experimentally determined map is free from any bias that may be introduced into a molecular-replacement-based model. Here, we expand the protocol to MR-SAD to allow (re)building from a potentially biased and incomplete model obtained by molecular replacement, computational modelling or any other external source. The new MR-SAD algorithm combines the information from an initial partial model with SAD data and density modification to extend the limits of successful low-resolution structure solution. Current methods (de La Fortelle *et al.*, 1997 ▸; Panjikar *et al.*, 2009 ▸; Adams *et al.*, 2010 ▸) represent and transfer phase information using Hendrickson–Lattman coefficients (Hendrickson & Lattman, 1970 ▸), where the phase information estimated from the constantly changing macromolecular and anomalous scatterer substructure models is statically passed between the different steps of the structure-solution process. Furthermore, current methods assume independence (Read, 1997 ▸) of the Hendrickson–Lattman coefficients when combining phase information. Our algorithm overcomes these shortcomings and provides phase estimates by simultaneously taking into account the diffracted intensities, macromolecular and substructure models, and the modified electron density, while modelling the errors in both the current structural models and the experimental data on which these are based. Furthermore, the new MR-SAD algorithm uses likelihood-based gradient maps (de La Fortelle *et al.*, 1997 ▸) to find any missing anomalous atoms at any step in the structure-solution process. We have applied the new algorithm to a number of low-resolution (3.0–4.5 Å) single-wavelength anomalous diffraction data sets for which incomplete partial models were available and we automatically obtained solutions that have evaded experts using other methods.

## 2. Methods   

**Figure 1:**
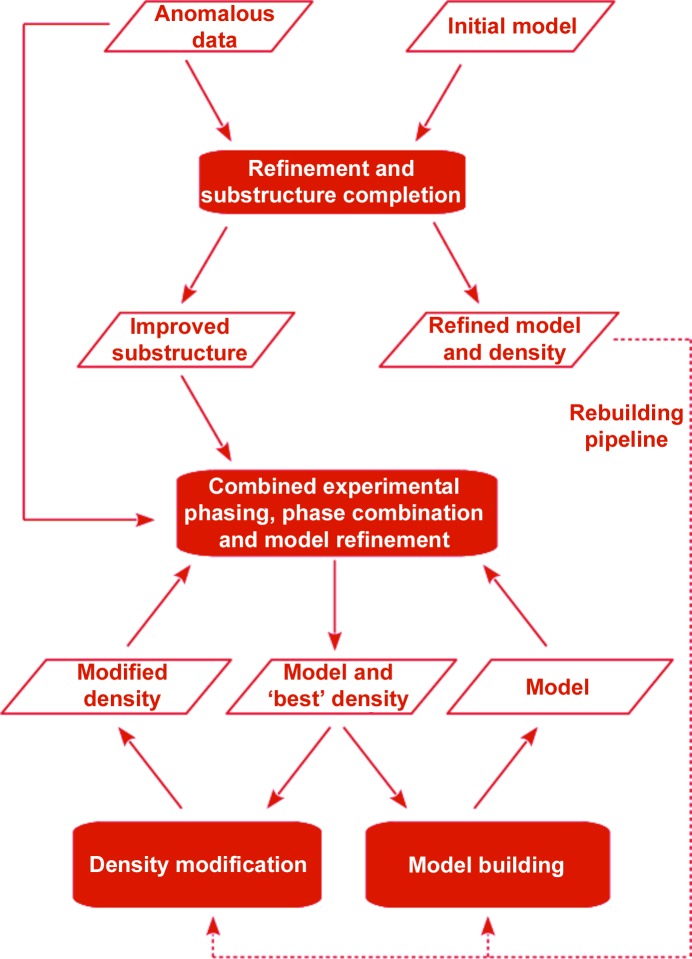
Flow chart for the ‘substructure-only’ and ‘rebuilding’ pipelines.

Two MR-SAD pipelines (Fig. 1 ▸) have been implemented in the *CRANK*2 (Skubák & Pannu, 2013 ▸) structure-solution software to simultaneously combine information from a partial model and anomalous scattering in the structure-solution process. The pipelines differ in how much information is used from the starting model that is typically obtained by molecular replacement. The source code implementing the pipelines and the multivariate function described in the following sections is released as an open source.

### 2.1. Pipelines and algorithm   

The ‘rebuilding’ pipeline uses the refined MR model, as shown by the dashed line in Fig. 1 ▸, the improved anomalous substructure and the ‘best’ (Blow & Crick, 1959 ▸) MR-derived electron density for subsequent rebuilding and model improvement with the ‘combined’ experimental phasing, phase-combination and model-refinement algorithm. The ‘substructure-only’ pipeline removes any possible MR-inherited protein model bias: the MR model is only used to improve the anomalous substructure and only the anomalous substructure is input into the ‘combined’ algorithm.

Both pipelines start with multiple iterations of refinement of the input partial model using the *REFMAC* SAD log-likelihood function (Skubák *et al.*, 2004 ▸) and detection of additional anomalous scatterers from SAD log-likelihood gradient maps (denoted as ‘Refinement and substructure completion’ in Fig. 1 ▸). If none or a very small portion of the anomalous substructure is present in the initial model, anomalous scatterers are detected using anomalous difference maps in the first iteration, followed by SAD log-likelihood gradient map detection in the following iterations. Furthermore, substructure atoms with occupancies refined below a certain threshold are removed from the substructure model.

For the rebuilding pipeline, the best electron density obtained from the refinement and substructure-improvement step is used both for initial rebuilding of the refined model and as input for crystal-space density modification. The rebuilt model, the modified map and the substructure are then all used by the combined experimental phasing, phase-combination and model-refinement function. The function provides a refined model and the actual best map that are used in the next iterations of crystal-space density modification and model building.

For the substructure-only pipeline, only the improved substructure is input into the initial iteration of the combined function, along with the data. In this special case, the function reduces to the experimental phasing SAD likelihood function that generates an ‘experimental’ electron-density map: an ‘unbiased’ map phased solely from the substructure and the SAD data. The experimental density is then input to density modification and the resulting modified map is used by the SAD phase-combination function, skipping the model-building branch of the algorithm in this iteration. Finally, the resulting best density is used for density modification and initial model building and the ‘combined’ algorithm procedure is iterated for the specified number of cycles.

### 2.2. Multivariate likelihood function   

The core of the MR-SAD pipelines consists of the ‘combined’ model-building function that simultaneously considers the anomalous substructure, the phase-improved electron density and the partial model, and dynamically refines the protein and anomalous substructure models using a maximum-likelihood treatment based on multivariate probability distributions: 
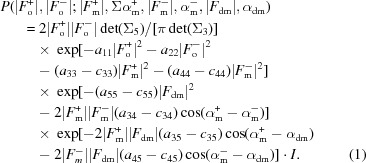



In (1), *I* is an integral (2) containing the unknown α_o_^−^ phase term: 
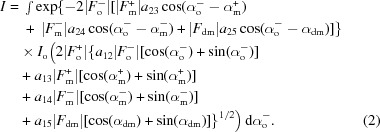



We are unaware of an analytical solution to the above integral, so our algorithm evaluates the integral numerically. The gradient of the logarithm of this general function is used to determine anomalous atoms that are currently missing (*i.e.* log-likelihood gradient maps) and thus considers information from the diffraction data, the current anomalous and non-anomalous atomic coordinates, phasing, density modification and model building all together. Determining missing anomalous atoms is often essential for MR-SAD, since incomplete molecular replacement models often do not contain all of the anomalous scatterers and a complete anomalous model can substantially improve the electron density.

In the above equations, |*F*_o_^+^|, |*F*_o_^−^| are the observed structure-factor amplitudes for the two reflections in a Bijvoet/Friedel pair, while |*F*_m_^+^|, α_m_^+^, |*F*_m_^−^|, α_m_^−^ are the structure-factor amplitudes and phases for the current model including anomalously scattering atoms and |*F*_dm_|, α_dm_ are the structure-factor amplitude and phase after density modification. *a*_*ij*_ is the *ij*th element of the inverse of the full 5 × 5 covariance matrix Σ_5_ for all structure factors (*F*_o_^+^, *F*_o_^−^, *F*_m_^+^, *F*_m_^−^, *F*_dm_), while *c*_*ij*_ is the *ij*th element of the 3 × 3 submatrix Σ_3_ of Σ_5_ consisting of the model structure factors (*F*_m_^+^, *F*_m_^−^, *F*_dm_). The Σ_5_ covariance matrix comes from a multivariate Gaussian complex distribution of structure factors: the starting point for the derivation of the distribution shown in (1) and (2). It not only contains information about the correlation between all involved structure factors, but can also incorporate refinable error terms. For example, the covariance-matrix element *a*_13_ = 〈*F*_o_^+^(*F*_m_^+^)*〉 = *D*(Σ_*j*_ *f_j_ + f_j_*′′), where the summation is over all *j* atoms in the unit cell and *f_j_* and *f_j_*′′ are the atomic scattering factors. *D* is a Luzzati parameter (Luzzati, 1952 ▸) that can account for errors in an incomplete model and also for model bias, and thus shows how information from a molecular replacement starting model can be incorporated and optimized by the likelihood function.

### 2.3. Structure-solution setup   

*CRANK*2 v.2.0.137 was used to run all of the jobs. Table 1 ▸ gives information on all of the diffraction data, the partial models and the anomalous scatterer(s). The diffraction data, the partial model and the anomalous scatterer(s), along with the protein sequence and the scattering factors (*f*′ and *f*′′), were all input into *CRANK*2. The number of molecules in the asymmetric unit, obtainable from the molecular-replacement solution, was also input for data sets where the default estimation from Matthews coefficients incorrectly estimated this number. Furthermore, all of the *CRANK*2 jobs were started with five refinement and substructure-improvement cycles instead of the current *CRANK*2 default of three, and a minimum of 15 cycles of combined model-building cycles instead of the default of five.

The combined function, implemented in the program *REFMAC*5 (v.5.8.0155; Murshudov *et al.*, 2011 ▸), was used by *CRANK*2 for all of the reciprocal-space refinement, phasing and phase combination. Furthermore, *CRANK*2 used *Parrot* (v.1.0.4; Cowtan, 2010 ▸) for crystal-space density modification and *Buccaneer* (Cowtan, 2006 ▸; v.1.6.3 was used for all data sets apart from the SecYEG–SecA complex, where the older v.1.6.1 was used owing to a weak density-filtering regression) for model building. Density modification by *Parrot* included solvent flattening, histogram matching and, for data sets with multiple monomers in the asymmetric unit, automatic NCS operator determination and NCS averaging. NCS averaging was not performed for F_1_-ATPase as the subunits are in different conformations. The programs *MOLREP* (Vagin & Teplyakov, 2010 ▸) and *Phaser* (McCoy *et al.*, 2007 ▸) were used to obtain the initial MR models. All these programs form part of the *CCP*4 suite (v.7.0.020; Winn *et al.*, 2011 ▸). *CRANK*2 is generally available from the *CCP*4 website (http://www.ccp4.ac.uk) and is best run from the new *CCP*4*i*2 graphical user interface (Potterton *et al.*, 2018 ▸).

For the SecYEG–SecA complex data set, a resolution cutoff of 7.0 Å and a lower r.m.s. threshold (4.25σ rather than the default 4.75σ) were used in the substructure-completion and refinement step. These adjustments of the default *CRANK*2 parametrization were not needed for any of the other data sets and could be specific to very low-resolution data.

## 3. Results   

**Figure 2:**
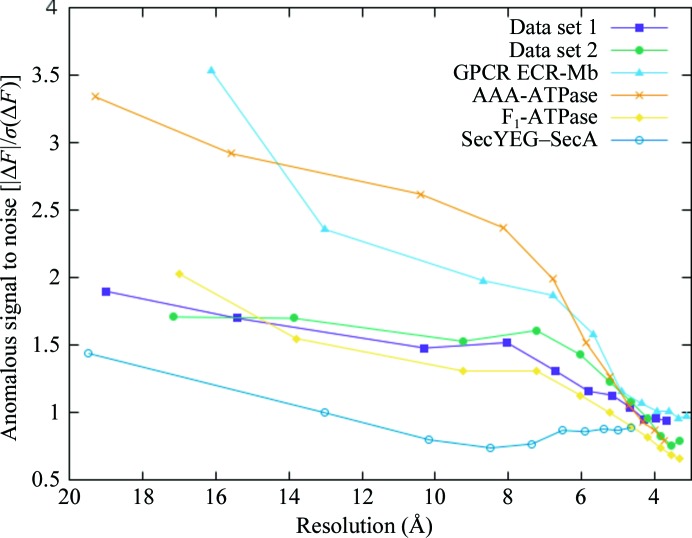
The anomalous signal-to-noise ratio *versus* resolution for all data sets.

Here, we show the results of the pipelines on six low-resolution data sets from six different proteins, each with relatively weak anomalous signals, containing crystallographic anomalies and/or with only incomplete molecular-replacement models available (Table 1 ▸). A plot of the anomalous signal-to-noise ratio {the absolute value of the Bijvoet difference divided by its standard deviation [|Δ*F*|/σ(Δ*F*)]} as a function of resolution for all data sets is shown in Fig. 2 ▸. The poor quality of the initial models for all data sets is indicated by *R*_free_ values of around 50% (Table 2 ▸).

### 3.1. Novel low-resolution structures determined   

‘Data set 1’, ‘data set 2’ and the extracellular region of the adhesion G protein-coupled receptor in complex with a monobody (Salzman *et al.*, 2016 ▸; GPCR ECR–Mb) represent novel crystal structures where highly complete models have been automatically produced and refined with our new method, but were not solved by the other multiple automatic and manual approaches that were tested. The identities of data sets 1 and 2 are withheld since the crystal structures derived from these have not yet been published.

The crystals that gave data sets 1 and 2 both exhibited translational noncrystallographic symmetry (tNCS), a crystallographic anomaly in which two or more molecules are in similar orientations in the asymmetric unit, resulting in systematically strong and weak diffraction intensities that often complicate structure solution and refinement. The crystal structure from data set 1 was solved in a unit cell containing tNCS, while an approximation that the tNCS is modelled by crystallographic translational symmetry turned out to be a more successful strategy for the solution of the crystal structure from data set 2. The diffraction data in data set 2 also suffered from radiation damage, as observed by a decrease in the observable diffraction limit during data collection. The structure solution, which was carried out during the 2016 CCP4/APS Crystallographic School (http://www.ccp4.ac.uk/schools/APS-2016/), proved that the data set was particularly challenging. Using either the substructure-only or the rebuilding pipeline, both structures have been clearly built, as seen from the low *R*_free_ values for the final models (Table 2 ▸).

An MR solution for the GPCR ECR–MB complex was found with a search model obtained using the Rosetta energy function (DiMaio *et al.*, 2011 ▸), as search models from the PDB failed to provide an MR solution. Noncrystallographic symmetry averaging could not aid in structure solution. Both pipelines in the current version of *CRANK*2 successfully built the model in just a few model-building iterations.

The structure of the vacuolar protein sorting 4 AAA-ATPase (Caillat *et al.*, 2015 ▸) complex is also a novel structure solved by our new algorithm from a diffraction data set extending to 3.6 Å resolution. The ability of *CRANK*2 to improve the *R*_free_ from 48 to 39% was important in solving the structure. Unlike for the other data sets, the quality of the protein model was already crucially improved in the first step of the pipeline, which consisted of SAD refinement iterations that added or replaced 34 of the total of 42 selenium-substructure atoms.

The above four novel test cases exhibit the power of the new algorithm to obtain a solution when other methods fail. The last two test cases are examples of previously solved structures that show the anomalous signal and resolution limits of the two pipelines.

### 3.2. Long-wavelength sulfur-SAD F_1_-ATPase data sets   

The structure of bovine mitochondrial F_1_-ATPase was initially solved after a time-consuming search to obtain a suitable heavy-atom derivative isomorphous to the native crystal (Abrahams *et al.*, 1994 ▸). An attractive alternative to avoid the problem of searching for heavy-atom derivatives is to use the intrinsic sulfur signal and merge data from multiple isomorphic crystals (Liu *et al.*, 2012 ▸). Long-wavelength sulfur-SAD data sets from multiple crystals of bovine mitochondrial F_1_-ATPase were collected at 6 keV and merged to produce a high-multiplicity data set extending to 3.0 Å resolution. We were unable to determine the positions of the over 70 intrinsic weakly anomalous S and phosphate atoms contained in the crystal structure using substructure-detection programs on the diffraction data alone, despite a systematic search through a large number of trials in a wide resolution-cutoff range (3.0–6.0 Å) and various other *ad hoc* optimization attempts. However, on inputting just the trimeric α chain from a 6.0 Å resolution F_1_-ATPase model (Sanchez-Weatherby *et al.*, 2009 ▸) obtained by molecular replacement, *CRANK*2 could find the anomalous substructure and the substructure-only pipeline built a nearly complete model to an *R*_free_ of 34.6%. To our knowledge, this represents the largest sulfur-SAD structure solved just from the SAD data and the positions of the anomalous substructure.

### 3.3. SecYEG–SecA SAD data set at 4.5 Å resolution   

Crystals of the SecYEG–SecA protein-translocation complex (Zimmer *et al.*, 2008 ▸) from *Thermotoga maritima* diffracted to 4.5 Å resolution and the resulting data set contained anomalous signal from selenomethionine-derivatized SecYEG. The authors originally solved the structure by molecular replacement, NCS and cross-crystal averaging, experimental MAD phases from selenomethionine-derivatized SecYEG and interative manual model building and refinement. To test our new MR-SAD protocol, we started from a molecular-replacement solution obtained from the 7.5 Å resolution structure of *Aquifex aeolicus* SecYEG and *Bacillus subtilis* SecA by the same authors (PDB entry 3dl8) that resulted in an *R*_free_ factor of 51.8% after ‘jelly-body’ refinement in *REFMAC*5 (Murshudov *et al.*, 2011 ▸). Despite the very low-resolution data and the poor starting model, both pipelines could automatically build the structure to an *R*_free_ of less than 40%.

## 4. Discussion   

For low-resolution data sets with a poor starting model, it appears to be essential to have an accurate and continually updating representation of the anomalous and non-anomalous model and an accurate error model and to combine the information from phasing, density modification and model building simultaneously. We believe that this is the reason that our method is able to succeed in these challenging cases.

In all of the reported structure determinations, both pipelines performed equally well, as judged by the *R*_free_ values of the models obtained (Table 2 ▸), with only small differences observed for all of the data sets. However, substantial differences between the pipelines could be observed in some cases if suboptimal parameters were used in data processing or structure solution: for example, only the rebuilding pipeline succeeded in building the structure from ‘data set 2’ processed at 3.0 Å resolution rather than 3.2 Å. Therefore, we suggest running both of the pipelines simultaneously for highly challenging data sets.

Intuitively, the advantage of the substructure-only pipeline is that the structures built can be considered to be free of bias from the starting molecular-replacement model. However, the correlations between the initial MR density map and the density maps from both *CRANK*2 pipelines were approximately the same in all cases. This suggests that at least for these test cases a larger number of cycles of the rebuilding pipeline can remove the bias from the initial MR model.

Future work may exploit models from the substructure-only pipeline to efficiently combine the results with the models from the rebuilding pipeline and the starting model itself to further improve the structure-solution process: combination of models from different structure-solution methods has already been shown to be effective (van den Bedem *et al.*, 2011 ▸).

Finally, the pipelines discussed here not only present an immediate solution to structural biologists attempting to solve structures from low-resolution X-ray data sets, but provide a mathematical framework that can be applied to free-electron laser data, three-dimensional macromolecular electron crystallography or electron microscopy. For example, the algorithm can be adapted for native sulfur-SAD phasing from X-ray free-electron data (Batyuk *et al.*, 2016 ▸). Yet, since the algorithm is general and uses the observed data and measurement errors directly together with combining all steps in structure solution, the full power of the method can be exploited if the errors and unmerged observed data are considered for the different experiments rather than assuming the data and error model from X-ray crystallography.

## Figures and Tables

**Table 1:** Crystal and molecular-replacement model statistics

	Final PDB code	Resolution (Å)	Anomalous scatterer(s)	No. of residues	Correct MR residues† (%)	Incorrect MR residues† (%)	R.m.s.d., correct residues (Å)
Data set 1	‡	3.6	Se	800	42.5	23.5	1.6
Data set 2	‡	3.2	Se	378	60.8	12.9	1.7
GPCR ECR–Mb	5kvm	3.0	I	459	49.7	11.7	1.5
AAA-ATPase	4d80	3.6	Se	1776	75.2	22.1	1.7
F_1_-ATPase	2w6f	3.0	S, P	3587	46.7	2.3	0.9
SecYEG–SecA	3din	4.5	Se	2886	47.9	40.7	1.7

† For the initial MR models, a residue is considered to be ‘correct’ if its C^α^ position is at most 4 Å distant from a deposited (or best known for data sets 1 and 2) C^α^* position and at least one of the C^α^ neighbours is at most 4 Å distant from a C^α^* neighbour. All other residues, *i.e.* residues not satisfying these criteria, are considered to be ‘incorrect’. The percentages are relative to the total number of residues.

‡ The refined models for data sets 1 and 2 have not yet been deposited in the PDB.

**Table 2:** *R*_free_ values for models after molecular replacement and after the automated *CRANK*2 pipelines

	Molecular-replacement solution†	Substructure-only pipeline‡	Rebuild pipeline‡
Data set 1	49.8	32.6	29.8
Data set 2	53.7	28.9	32.3
GPCR ECR–Mb	48.6	39.1	38.4
AAA-ATPase	47.5	39.0	40.9
F_1_-ATPase	46.5	34.8	33.8
SecYEG–SecA	51.8	39.9	39.6

† The *R*_free_ values after *REFMAC*5 ‘jelly-body’ refinement of the molecular-replacement solution using 50, 75 or 100 refinement cycles, whichever provided the best value.

‡ The *R*_free_ values after *REFMAC*5 ‘jelly-body’ refinement of the model output by *CRANK*2 using an additional 0, 25 or 50 refinement cycles, whichever provided the best value.
